# Bison, Elk, and Other Captive Wildlife Species Humoral Immune Responses against SARS-CoV-2

**DOI:** 10.3390/ani14192829

**Published:** 2024-09-30

**Authors:** Mehrnaz Ardalan, Konner Cool, Natasha N. Gaudreault, Dashzeveg Bold, Catherine Rojas, Anna Mannix, Janine Seetahal, Juergen A. Richt, Roman M. Pogranichniy

**Affiliations:** 1 Department of Diagnostic Medicine/Pathobiology, College of Veterinary Medicine, Kansas State University, Manhattan, KS 66506, USA; ardalan@ksu.edu (M.A.); nng5757@vet.k-state.edu (N.N.G.); jricht@vet.k-state.edu (J.A.R.); 2 Veterinary Diagnostic Laboratory, Department of Diagnostic Medicine/Pathobiology, College of Veterinary Medicine, Kansas State University, Manhattan, KS 66506, USA

**Keywords:** bison, elk, wildlife species, SARS-CoV-2, surveillance, ELISA

## Abstract

**Simple Summary:**

Severe acute respiratory syndrome coronavirus 2 (SARS-CoV-2), the virus responsible for COVID-19, primarily spreads among humans, but there have been cases of transmission between humans and animals, as well as some instances of animal-to-animal transmission. Several zoos have reported cases of large cats such as tigers and lions, gorillas, and other animal species testing positive for SARS-CoV-2, presumably due to contact with humans. White-tailed deer (WTD) are highly susceptible to SARS-CoV-2 and are prevalent throughout the United States with a high population density. Separately, there is limited research and reporting on the susceptibility of bison, elk, and other wildlife species to SARS-CoV-2. This study determines the SARS-CoV-2 seroprevalence for various captive wildlife, elk, and bison, across different regions in the United States, using several serological tests. The presence of neutralizing antibodies to the virus in cheetahs, gorillas, lions, hippopotamuses, elk, and bison indicates that they are susceptible to SARS-CoV-2 infection. This highlights the importance of continuous monitoring of the prevalence of SARS-CoV-2 antibodies in various species that come into close contact with humans.

**Abstract:**

Severe acute respiratory syndrome coronavirus 2 (SARS-CoV-2), the virus responsible for COVID-19, has been found to infect various domestic and wild animal species. In this study, convenience serum samples from 575 bison, 180 elk, and 147 samples from various wildlife species collected between 2020 and 2023 from several regions in the United States were analyzed for the presence of SARS-CoV-2-specific antibodies. Two commercial ELISA assays based on the inhibition of the SARS-CoV-2 receptor-binding domain (sVNT) or the nucleocapsid protein (N-ELISA) of SARS-CoV-2 were used. Positive samples from the sVNT were additionally evaluated using a conventional virus neutralization test (VNT). Our results indicated that 1.2% of bison, 2.2% of elk, and 4.1% of the other wildlife species serum samples were seropositive in the sVNT, whereas 4.2% of bison, 3.3% of elk, and 1.4% of the other captive wildlife species serum samples tested positive by the N-ELISA. Among the sVNT serum samples, two samples from bison, one sample from elk, and five serum samples from other wildlife species (one cheetah, one gorilla, two lions, and one hippopotamus) had neutralizing antibody titers in the VNT, indicating these species are susceptible to SARS-CoV-2 infection. These findings highlight the importance of broad surveillance efforts for the effective monitoring of SARS-CoV-2 in non-human hosts.

## 1. Introduction

Zoonotic pathogens account for 75% of emerging infectious diseases [1] and represent a significant threat to global economic stability and public health. Humans, animals, and the environment greatly influence the emergence and transmission of various infectious diseases. Over recent decades, newly emerged diseases in humans have originated from animals due to the consumption of animal-derived food products [2]. Wild animals serve as reservoirs for numerous infectious diseases with zoonotic potential. A significant percentage (72%) of zoonotic infections originate from wildlife [3]. Humans face increased risks of contracting these diseases due to heightened interaction with wild animals [1]. Accordingly, an increase in cross-species virus infection and zoonotic transmission can be a result of growing human–animal interaction through wet markets, exotic pet trade, hunting, and farmed animals [4,5]. Therefore, it is necessary to identify those pathogens and the host species and determine the circumstances most likely to be the source of creating reservoirs for infection.

The vast majority of animal SARS-CoV-2 infections result from human sources, especially among domestic and captive animal populations. Transmission to free-living wildlife is less common and less understood due to fewer opportunities for direct contact [6]. However, certain activities such as animal rescue, conservation efforts, and wildlife tourism can elevate the risk of transmission. Indirect transmission pathways may also exist, including human contamination of the environment (anthropogenic food waste) and supplemental food used for hunting and/or wildlife viewing [6].

SARS-CoV-2 is a single-stranded, positive-sense, enveloped RNA virus belonging to the Coronaviridae family, genus Betacoronavirus [7]. Analyzing the interaction between (S) RBD:ACE2 has made it possible to predict potential susceptible host species for SARS-CoV-2 infection, predicting a wide range of mammals [6]. The high immunogenicity of the RBD contributes to the induction of cellular immunity against SARS-CoV-2, making it a key target for neutralizing antibodies [8,9].

The N protein is one of the main immunogenic proteins of SARS-CoV-2, making it an ideal target for detection in serological assays. However, the high conservation of the N protein among related coronaviruses [10] could result in cross-reactions and false positives in diagnostic tests [11].

The susceptibility of animals to SARS-CoV-2 varies, and different species have different levels of susceptibility. Captive cats (including pumas, cougars, snow leopards, lions, and tigers), white-tailed deer, and various other wildlife in captivity, such as gorillas and otters, have been affected [12]. As of June 2023, the epidemiological data from the World Organization for Animal Health (WOAH) reported 775 animal outbreaks worldwide, affecting 29 species in 36 countries [13]. These species include domestic pets (such as dogs, cats, ferrets, and hamsters), zoo inhabitants (including large cats, otters, and gorillas), farmed animals (such as mink), and wildlife (such as white-tailed deer) [13]. Several animal species, such as non-human primates, white-tailed deer, ferrets, Syrian golden hamsters, cynomolgus macaques, and raccoon dogs, have been shown to be susceptible to SARS-CoV-2 following experimental inoculation, and they are capable of shedding and/or transmitting the virus to other animals [14,15,16,17,18,19,20]. Dogs, sheep, and cattle exhibit limited susceptibility, and swine as well as avian species such as chickens and ducks demonstrate resistance to infection [20]. Factors such as adequate cell receptors, body temperature, population density, group behavior, and human–animal interactions play crucial roles in determining the potential for animals to host and spread SARS-CoV-2 [21].

There are several reports that provide evidence of a reverse zoonosis with SARS-CoV-2 between mink and humans in mink-farm workers in the Netherlands [22] and between hamsters and humans [23]. Rapid virus transmission within the mink population has led to the emergence of a new mink-associated variant of SARS-CoV-2, which has been identified in both humans and mink [22]. More commonly, SARS-CoV-2 has spilled over from infected humans into animals. In January 2021, several gorillas at the San Diego Zoo Safari Park tested positive for SARS-CoV-2 [24]. The gorillas likely contracted the virus from an asymptomatic staff member who tested positive for the virus. The affected gorillas exhibited symptoms consistent with respiratory infection, such as coughing, congestion, and mild lethargy, similar to those seen in humans [24]. This incident raised concerns about the susceptibility of great apes, including gorillas, orangutans, and chimpanzees, to the virus. Following that, SARS-CoV-2 was also detected in gorillas in a zoo in Prague, Czech Republic [25], and in Rotterdam Zoo, the Netherlands [26]. Researchers have suggested that non-human primates such as gorillas and big cats can be infected with SARS-CoV-2, develop clinical signs, and effectively spread the disease [25].

The abundance and geographic distribution of cervid species, such as the white-tailed deer, demonstrates that they are free-living wild animals widespread in Northern and Eastern regions of North America, particularly due to their close proximity to human dwellings [27]. White-tailed deer have been identified as potentially susceptible to SARS-CoV-2 infection due to the high similarity between their ACE2 sequence and that of humans [28]. A recent experimental study conducted by Porter et al. [29] investigated the susceptibility of weanling elk (*Cervus canadensis*) and mule deer (*Odocoileus hemionus*) to SARS-CoV-2. The study revealed weanling elk showed low susceptibility to infection with the delta variant of SARS-CoV-2. However, they did develop low-level antibody responses. Conversely, mule deer were found to shed infectious virus capable of infecting naïve mule deer and developed high-level antibody responses. This indicates that mule deer have the potential to play a role in SARS-CoV-2 epidemiology [29]. Another experimental study demonstrated that North American elk, including elk calves and adults, not only developed virus-neutralizing antibodies but also had viral RNA detected in the medial retropharyngeal lymph node [30], suggesting that North American elk are susceptible to infection with the ancestral Wuhan-like variant of SARS-CoV-2 (USAWA1/2020) [30].

Bison (*Bison bison*) and buffalo (*Bubalus bubalis*) are a known reservoir species for some coronaviruses, such as BCoV [31]. However, the susceptibility of bison or buffalo to SARS-CoV-2 and the potential consequences are not well-documented. In a systematic surveillance study, Asian buffaloes tested positive for SARS-CoV-2 RNA, suggesting that buffaloes are susceptible to SARS-CoV-2 infection [32]. Ahmed et al. [33] identified a potential risk of SARS-CoV-2 infection among cattle, buffalo, goats, and sheep, which are the main species in family farming systems in South Asia.

Zoos provide a unique environment to document reverse zoonotic transmission events for observing and studying human–animal interactions due to the close proximity between a wide variety of wildlife species and animal caretakers. These settings increase the chances for sporadic cross-species transmission and raise concerns about the potential for widespread infection among animals that interact closely with humans. Natural infections of captive feline species in zoos have revealed that they are highly susceptible to SARS-CoV-2 [34]. Natural infection in captive cats has been demonstrated in Asia, Europe, and the Americas [20]. In March 2020, the first natural infections by SARS-CoV-2 were reported in tigers (*Panthera tigris*) and lions (*Panthera leo*), exhibiting only mild respiratory signs, at the Bronx Zoo in New York City, USA, following exposure to zookeepers [35]. Further reports list SARS-CoV-2 infections in tigers at Knoxville Zoo in Tennessee, USA [36]; Malayan tigers at a zoo in Virginia, USA [37]; Amur tigers, Canadian lynx, and African lions at the Pittsburgh Zoo, USA [38]; Asiatic lions, Bengal tigers, and leopards in India [39]; an African Lion at a zoo in Indiana, USA [40]; lions at the Barcelona Zoo, Spain [41], and at Rotterdam Zoo, the Netherlands [26]; Asiatic lions and Sumatran and Malayan tigers in a zoo in Prague, Czech Republic [25]; and snow leopards at the Louisville Zoo in Louisville, Kentucky, USA [42]. Additionally, natural infections by SARSCoV-2 have been reported in hippopotamuses at zoos in Hanoi (Vietnam) [43] and Belgium [44].

Following the emergence of SARS-CoV-2 in humans, likely originating from an animal host, there has been considerable interest in exploring the potential of animals as reservoirs for SARS-CoV-2. Experimental infection studies and surveillance efforts (molecular and serological) suggest a broad host range of SARS-CoV-2, particularly those in close contact with humans, have been infected with SARS-CoV-2. Although extensive studies on the virus’s susceptibility in bison and elk have not been conducted, it is crucial to consider the possible risks, particularly in situations where there is human–animal interaction, such as in captive settings or areas where animals and humans coexist closely. The primary aims of this research were to investigate the seroprevalence of SARS-CoV-2-specific antibodies in bison, elk, and other wildlife species across different regions of the United States using two commercially available ELISAs designed to detect antibodies specific to the nucleocapsid (N) and spike (S) proteins of SARS-CoV-2. Additionally, we evaluated the performance of serological detection methods for identifying SARS-CoV-2 antibodies in these animal populations.

## 2. Materials and Methods

### 2.1. Sample Collection

**Figure 1:**
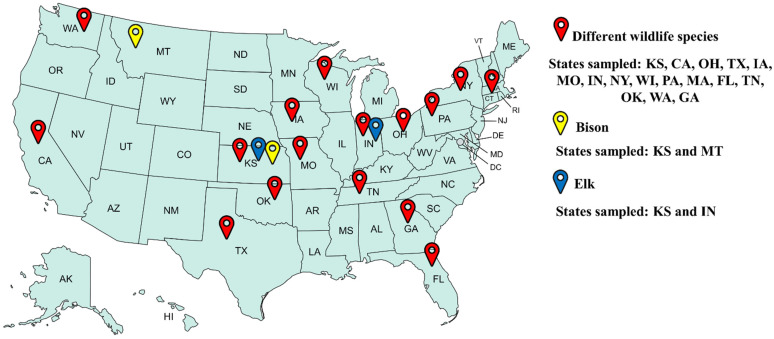
Map showing the distribution of serum samples collected from bison, elk, and different captive wildlife species (as mentioned in Materials and Methods) across the United States (created with https://mapchart.net).

Convenience serum samples provided by Kansas State Veterinary Diagnostic Laboratory consisted of 575 bison serum samples collected between 2020 and 2022 from two states [Kansas (KS) and Montana (MT)]; 199 elk samples collected in 2016, 2022, and 2023 from two states [Indiana (IN) and Kansas (KS)]; and 147 serum samples from several captive species (elephant, gray fox, fox, llama, camel, koala, alpaca, rhinoceros, lion, addax, giraffe, panda, tiger, goral, porcupine, oryx, cheetah, zebra, hippopotamus, wallaby, cockatiel, macaw, bearcat, eagle, lynx, chevrotain, mink, sea lion, lar gibbon,, antelope, caribou, bobcat, hyena, impala, primate, ferret, bear, buck, and gorilla) collected between 2020 and 2023 from sixteen U.S. states [Kansas (KS), California (CA), Ohio (OH), Texas (TX), Iowa (IA), Missouri (MO), Indiana (IN), New York (NY), Wisconsin (WI), Pennsylvania (PA), Massachusetts (MA), Florida (FL), Tennessee (TN), Oklahoma (OK), Washington (WA), and Georgia (GA)]. These samples were analyzed for the presence of SARS-CoV-2-specific antibodies (Figure 1; Table 1). Upon arrival, all samples were cataloged according to the owner’s location. Samples lacking owner location data were cataloged based on location of the veterinary clinic instead.

### 2.2. Detection of SARS-CoV-2-Specific Antibodies by ELISAs

#### 2.2.1. SARS-CoV-2 Double Antigen ELISA for Detection of N-Specific Antibodies (N-ELISA)

Serum samples were tested for SARS-CoV-2 N-specific antibodies using the commercially available ID Screen^®^ SARS-CoV-2 Double Antigen Multi-species ELISA (Innovative Diagnostics, Grabels, France), referred to here as the N-ELISA, according to the manufacturer’s instructions. Briefly, serum samples were heat-inactivated at 56 °C for 30 min. Each well of the ELISA plate then received 25 μL of dilution buffer, followed by the addition of 25 μL of serum. Positive and negative assay controls were included with each run. The mixture was then incubated for 45 min at 37 °C. After incubation, each well was washed 5 times with 300 μL of wash solution. Following the wash, 100 μL of N protein recombinant antigen horseradish peroxidase (HRP) conjugate was added and incubated at room temperature (RT) for 30 min. Subsequently, the wells were washed 5 times with 300 μL of wash solution. After this, 100 μL of substrate solution was added to each well and incubated for an additional 20 min at RT before the reaction was stopped by addition of 100 μL stop solution. The optical density (OD) was measured at 450 nm using an ELISA microplate reader (BioTek Cytation5; Agilent, Santa Clara, CA, USA) immediately afterward. The OD of each sample was calculated as the S/P percentage (S/P%). Serum with S/P% ≥ 60% was considered positive, while serum with S/P% 50−60% was defined as ‘suspect’, and serum with S/P% ≤ 50% was considered negative.

#### 2.2.2. SARS-CoV-2 Surrogate Virus Neutralization Test (sVNT)

The SARS-CoV-2 surrogate virus neutralization test (sVNT; GenScript L00847, Piscataway NJ, USA) was used for detection of neutralizing antibodies against the RBD of the virus S protein. The test was conducted according to the manufacturer’s instructions. Briefly, serum samples were heat-inactivated at 56 °C for 30 min. Serum samples and the positive and negative assay controls were each diluted 1:10 in sample dilution buffer. Subsequently, these diluted samples were mixed with an equal volume of HRP-conjugated RBD, which was diluted at 1:1000. The mixture was then incubated at 37 °C for 30 min. After incubation, 100 μL of each mixture was added to a plate that was precoated with human ACE2 protein, which binds with the viral RBD. Following incubation at 37 °C for 15 min, the plate was washed four times with 260 μL of wash solution. Subsequently, 100 μL of tetramethylbenzidine substrate (TMB) was added to each well and incubated at RT for 15 min before the reaction was stopped by addition of 50 μL stop solution. The absorbance was read at 450 nm (OD450) using an ELISA microplate reader (BioTek Cytation5; Agilent, Santa Clara, CA, USA) immediately afterward. The OD of each sample was calculated as the inhibition percentage (% inhibition). For the expression of the results, % inhibition ≥ 30% was considered positive, and % inhibition < 30% was considered negative.

### 2.3. Virus-Neutralizing Antibodies 

#### 2.3.1. SARS-CoV-2 Neutralization Assay (VNT)

The purpose of the SARS-CoV-2 neutralization assay was to confirm the presence or absence of SARS-CoV-2-neutralizing antibodies in samples that were positive or were chosen as negative (pre-pandemic) by the sVNT.

SARS-CoV-2-neutralizing antibodies in sera were determined using a microneutralization assay, as previously described [45]. Briefly, heat-inactivated (56 °C/30 min) serum samples were diluted and then subjected to 2-fold serial dilutions starting at 1:8 and tested in duplicate. SARS-CoV-2 virus stocks (USA/WA1/2020; BEI NR: 52281) were diluted to 100 TCID_50_ in 100 μL DMEM culture media (1000 TCID_50_/mL) and added 1:1 to 100 μL of the sera dilutions. The virus/sera dilutions were then incubated for 1 h at 37 °C. The mixture was subsequently transferred to 96-well plates seeded with a confluent monolayer of Vero-E6 cells stably expressing the transmembrane serine protease 2 (Vero-E6/TMPRSS2). The neutralizing antibody titer was recorded as the highest serum dilution, at which at least one of the wells showed complete virus neutralization based on the absence of CPE observed under a light microscope at 96 h post infection. Positive control sera and back-titrations of diluted virus stock were used to monitor assay performance and consistency.

#### 2.3.2. BCoV Neutralization Assay

A classic neutralization assay was used to determine if neutralizing antibodies against bovine coronavirus were present. Serum samples were first heat-inactivated at 56 °C for 30 min and were diluted 1:8 with Dulbecco modified Eagle medium (DMEM; Gibco™ 11965092, Grand Island, NY, USA) containing Trypsin (1000X, TRYPSIN-TPCK TREATED-IRRADIATED; Worthington, OH, USA) and Antibiotic-Antimycotic (100X; CORNING 30-004-Cl, Manassas, VA, USA). Subsequently, 100 μL of each serum dilution was combined with 100 μL of supplemented media on 96-well plates and subjected to 2-fold serial dilutions starting from 1:8 to 1:2560. In addition, back titration was performed for each viral dilution. The BCoV virus stock was diluted to 100 TCID_50_/100 μL, and then 100 μL of diluted virus in DMEM was added to 100 μL of the sera dilutions and incubated for 1 h at 37 °C. Following incubation, 100 μL of the virus–serum mixtures were transferred to 96-well plates containing confluent monolayers of human rectal tumor (HRT) cells and incubated for 48 h at 37 °C to allow for the infection of the cells. After incubation, the wells were washed two times with 0.01 M phosphate-buffered saline (PBS; pH 7.4) (PBS-T) and fixed with 80% acetone for 10 min. After acetone fixation, the plates were left under a fume hood for 5 h to dry. Following fixation, samples were rehydrated with PBS-T, and 50 μL of BCoV-specific primary antibody, Z3A5 (developed in-house), a monoclonal antibody that targets the spike protein subunit of BCoV, was diluted 1:10, added to each well, and incubated for 1 h at 37 °C. After 1 h, plates were washed two times with PBS-T, and 50 μL of Anti-Mouse IgG (H + L) secondary antibody (Jackson ImmunoResearch, West Grove, PA, USA, Code: 115-095-003), diluted 1:75, was added to each well and incubated for 1 h at 37 °C. Following incubation, plates were washed two times with PBS-T and observed under a fluorescence microscope (Nikon ECLIPSE TE2000-U, Nikon, Yokohama, Japan) to determine endpoint titer to BCoV based on the presence or absence of viral replication. Samples with known BCoV-neutralizing antibodies were used as a positive control and to monitor consistency between assays.

## 3. Results

**Figure 2:**
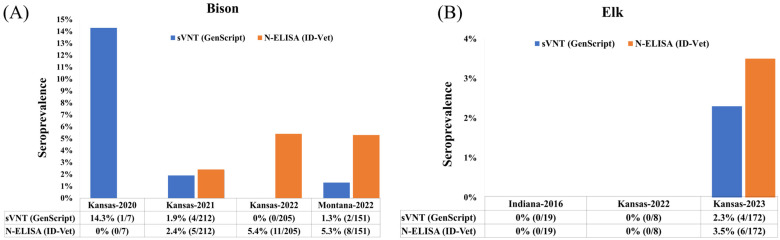
Detection of SARS-CoV-2-specific antibodies against the N and S (RBD) proteins in (**A**) bison and (**B**) elk using commercial N (ID-Vet; orange bars) and sVNT (GenScript, Piscataway NJ, USA; blue bars) IgG ELISAs. The *X*-axis represents the number and percentage of serum samples by collection year and location that were positive in the sVNT and N-ELISAs; the *Y*-axis represents the percentage of SARS-CoV-2 antibody seropositivity.

Of the 575 bison sera screened via sVNT, 14.3% (1/7) in 2020, 1.9% (4/212) in 2021, and 0% (0/205) in 2022 from Kansas, and 1.3% (2/151) in 2022 from Montana resulted as positive for detection of SARS-CoV-2 RBD. When the same samples were evaluated for the presence of SARS-CoV-2 nucleocapsid protein (N-ELISA), antibodies were detected in 0% (0/7) of bison sera collected in 2020, 2.4% (5/212) in 2021, and 5.4% (11/205) in 2022 from Kansas, and 5.3% (8/151) in 2022 from Montana, indicating higher seropositivity when evaluated with the N-ELISA compared to sVNT (Figure 2A). Sera from elk collected in 2016, before the COVID-19 pandemic, showed no antibodies against the RBD (sVNT) and nucleocapsid protein (N-ELISA). Out of the 180 elk serum samples screened by both sVNT and N-ELISA, 0% 0/8 samples from 2022 in Kansas tested positive. However, in 2023, the SARS-CoV-2 positivity rates in elk from Kansas were 2.3% (4/172) and 3.5% (6/172) for sVNT and N-ELISA, respectively (Figure 2B).

Out of the 147 serum samples collected from various wildlife species, 4.1% (6/147) tested positive for the sVNT assay. Among the sVNT-positive samples, positive antibody titers were identified in one cheetah from Massachusetts, one gorilla from Massachusetts, two lions from Kansas, and one hippopotamus from Massachusetts. The highest neutralization titers were found in lions (1:2048), followed by cheetah (1:512), gorilla (1:128), and hippopotamus (1:8) (Table 1). Antibodies against SARS-CoV-2 nucleocapsid protein (N-ELISA) were only detected in one gorilla and one goral, indicating a lower seropositivity rate of 1.4% (2/147) (Table 1). Notably, sera from different animal species that tested positive for antibodies to the RBD were not further examined to determine the presence of neutralizing antibodies against BCoV.

### Concordance among SARS-CoV-2 sVNT and VNT and BCoV Test in Bison and Elk

Among the eleven sVNT-positive samples, two samples from bison (titers 1:8 and 1:32) and one sample from elk (titer 1:16) showed SARS-CoV-2-neutralizing antibodies via VNT using the USA/WA1/2020 isolate. Of the seven bison and four elk serum samples that were positive for sVNT, neutralization activity against BCoV was observed in six bison (range 1:8–1:2048) and four elk (range 1:8–1:128). However, among the four sVNT- and BCoV-positive samples in elk, only one elk (titer 1:128) also tested positive by VNT (Table 2). In bison, among the 7 sVNT-positive samples and 57 randomly selected VNT- and sVNT-negative samples, 44 samples tested positive for BCoV. Higher seropositivity was observed when BCoV was positive and both sVNT and VNT results were negative (Table 2).

## 4. Discussion

The epidemiology of SARS-CoV-2, as well as its symptoms, morbidity, and mortality, has a wide range of variations around the world. When faced with an outbreak of novel zoonotic pathogens, such as SARS-CoV-2, it is critical that rapid diagnostic tools are developed, validated, and widely distributed to help inform policy decisions relating to animal and public health. There are several diagnostic methods available to evaluate the exposure of animals to SARS-CoV-2, including assays for the detection of antibodies, antigens, and molecular detection methods. It is important to note that there exists a range of diagnostic sensitivity and specificity in commercial assays, occasionally resulting in false negatives that can lead to a lack of effective intervention, possibly allowing for increased disease spread [46]. Moreover, false positives due to cross-reactivity pose significant challenges in diagnostic testing, as they can lead to incorrect diagnoses. Since SARS-CoV-2 and BCoV belong to the betacoronavirus family [47], there is a potential for cross-reactivity with the similar antigens of these betacoronaviruses, resulting in false positives. Therefore, it is essential to validate and verify the performance characteristics of diagnostic tests for species of interest and specificity to the pathogen of interest in order to minimize the risk of false positives and ensure the accuracy of the test results.

Infection with SARS-CoV-2 induces the host to produce detectable levels of IgG antibodies, which results in the development of protective immune responses and plays a crucial role in long-lasting immunity by eliciting a robust B-cell response [48]. An individual host’s immune response can be influenced by various factors, including previous exposure to infections and vaccinations, the severity of infections, and the other co-morbidity factors (weight, immunocompromised, age, and pulmonary conditions) [49,50]. The identification of clinical symptoms in captive animals in zoos, such as coughing, nasal discharge, lack of appetite, lethargy, and subsequent diagnostic investigations, has led to the identification of several species susceptible to natural infection [35,51]. However, subclinical infection of SARS-CoV-2 infections may remain undetected because many animals can carry pathogens without showing any clinical signs of illness. Additionally, wildlife and animals in inaccessible areas are less likely to be routinely monitored for clinical signs, allowing infections in these populations to go unnoticed and potentially spill over to domestic animals or humans. These infections may only be identified through laboratory tests or post-mortem examinations.

The present study aimed to assess the seroprevalence of SARS-CoV-2-specific antibodies in bison, elk, and different captive species from several geographically distinct regions in the United States to better understand the occurrence of natural infection by SARS-CoV-2 in these species. Additionally, this study assessed the diagnostic performance of two commercial ELISA kits in detecting antibodies against the SARS-CoV-2 N and S proteins in several species. A VNT was used as a reference test to confirm the results obtained from the sVNT. There have been reports of natural SARS-CoV-2 infection in captive wildlife species such as captive cats (tiger, lion, etc.), semi-aquatic mammals (hippopotamus), and other non-human primates (gorilla), suggesting that they are highly susceptible to the virus.

In Belgium [44], a study screened for the potential circulation of SARS-CoV-2 in two adult Hippopotamus amphibius using ELISA Wantai (RBD-ELISA), ID Screen^®^ SARS-CoV-2 Double Antigen (N-ELISA), and polymerase chain reaction (PCR). Nasal discharge was observed in both hippopotamuses. The results showed the presence of SARS-CoV-2 RNA in 53% of pool water samples and 27% of the combined nasal and fecal samples, which was suspected to have been transmitted to the hippopotamuses by asymptomatic zookeepers. Neutralizing antibodies against SARS-CoV-2 were detected with Wantai (16.79%) and ID Screen (60.33%), suggesting that hippos had developed specific immune responses after an active infection [44]. Following the detection in hippopotamus, additional studies [52] were conducted to evaluate possible SARS-CoV-2 infections in mammals, including those that could have been in indirect contact with the hippopotamuses, in two Flemish zoos between 2020 and 2021. Out of 50 collected serum samples from 26 mammal species and 1523 fecal samples from 103 mammal species, none of the samples tested positive by sVNT (GenScript cPass™, Piscataway NJ, USA) and PCR [52]. Another study reported the death of a hippopotamus due to SARS-CoV-2 infection 17 days after the onset of clinical symptoms in a zoo in Hanoi, which can be attributed to the close relation between the SARS-CoV-2 strain in hippopotamus and three human SARS-CoV-2 strains in Vietnam. Tissue samples taken from the lung, spleen, liver, and intestine tested positive by PCR [43]. In line with previous studies, we detected the presence of SARS-CoV-2 antibodies in hippopotamus serum samples. Together, these results indicate that hippopotamuses are susceptible to SARS-CoV-2 and should be among the species monitored for infection.

Incidences of SARS-CoV-2 infection have been reported in non-human primates and large cats in captivity at several locations across Europe and North America since 2020. Many of these cases have been linked to SARS-CoV-2-infected human caretakers. Both molecular and serological diagnostic methods have been successful in monitoring these events. In February 2021, at the Prague Zoological Garden, western lowland gorillas, Asiatic lions, Sumatran and Malayan tigers, and Amur leopards tested positive for the SARS-CoV-2 B.1.1.7 variant by RT-qPCR [25]. Almost all cases were symptomatic, presenting with clinical signs such as cough and nasal discharge. Among the zookeepers, one gorilla handler and two cat handlers tested positive for COVID-19 shortly before the outbreak in the animals, suggesting that SARS-CoV-2 transmission likely occurred via direct contact with infected humans [25]. Another multispecies animal outbreak occurred at Rotterdam Zoo in the Netherlands in November 2021. Fecal and nasal samples from seven western lowland gorillas living in the same enclosure and four Asiatic lions that developed symptoms were confirmed SARS-CoV-2 positive by RT-qPCR [26].

In October 2020, three Malayan tigers (Panthera tigris jacksoni) at a Tennessee zoo tested positive for SARS-CoV-2 by RT-qPCR, potentially linked to positive zookeepers. All the tigers exhibited symptoms and clinical signs [36]. Similarly, another study reported the presence of viral RNA in nasal swabs, nasal turbinates, lung tissue, and intestinal tissues in an African lion in Indiana zoo, USA [40]. In Barcelona Zoo in November 2020 [41], four lions showed respiratory clinical signs, and SARS-CoV-2 RNA was detected in nasal and fecal samples from the infected lions. Subsequent serological tests revealed that all four lions developed neutralizing antibody responses. Although high levels of neutralizing antibodies (nAbs) were initially observed with both SNT and sVNT, these levels decreased over a 4-month period following infection. When the serum was evaluated for the presence of SARS-CoV-2 N protein, low levels of anti-N antibodies were detected in three of the four tigers [41].

A study at the Virginia Zoo in the USA found that three Malayan tigers exhibiting respiratory symptoms were infected with the SARS-CoV-2 alpha (B.1.1.7) variant. A unique finding in this report was that no employees had tested positive for SARS-CoV-2 or exhibited any clinical symptoms in the four weeks prior to the detection in tigers [37]. Furthermore, Tewari et al. [38] reported five African lions, four Amur tigers, and a Canadian lynx at the Pittsburgh Zoo, USA, exhibited respiratory clinical signs and tested positive for viral RNA in feces. It was shown that lions were naturally infected with the alpha variant (B.1.1.7 lineage), and tigers and lynx tested positive for the delta variant (AY.25.1 lineage). A strong neutralizing antibody response against the viral spike protein was observed in lions, tigers, and lynx. However, none of the cheetahs showed any clinical signs, and antibody responses were not detected by surrogate virus neutralization assay, indicating the absence of exposure to SARS-CoV-2 or resistance to infection [38].

A serological study evaluated the prevalence of SARS-CoV-2 infection among 126 leopards, 96 Asiatic lions, and 98 Bengal tigers from eight Indian states. The results indicated the presence of SARS-CoV-2-neutralizing antibodies in 48 out of 320 serum samples, including 24 lions, 14 tigers, and 10 leopards, with titers (as the log of the reciprocal of the dilution in the range of <1.5, 1.8, 2.1, and >2.4) ranging from 2.1 to more than 2.4 [39]. Moreover, SARS-CoV-2-positive RT-qPCR results were reported in 2 out of 18 and 1 out of 20 Asiatic lions at two different locations in India [53]. A study conducted in Thailand during 2020–2021 [54] showed that 6.5% of captive tigers had SARS-CoV-2-neutralizing antibodies against the Wuhan Hu-1 and delta variants via the plaque reduction neutralization test (PRNT). The possible exposure to SARS-CoV-2 of tigers could be attributed to close interactions with their caretakers, who had a recent history of SARS-CoV-2 infection. However, only one seropositive tiger had a low level of neutralizing antibodies against the Omicron BA.2 subvariant [54].

In our study, SARS-CoV-2-positivity rates among the 147 captive wildlife species were 4.1% (6/147) for sVNT, including one Elephant, one hippopotamus, one gorilla, two lions, and one cheetah, and 1.4% (2/147) for N-ELISA, including one gorilla and one goral (Table 1). Lower seropositivity was observed when the same samples were evaluated with the N-ELISA. Samples that were positive for sVNT were subsequently evaluated using the VNT, and we observed that 4/6 sVNT-positive samples, including one gorilla, two lions, and one cheetah, were also positive for VNT.

The increases in SARS-CoV-2 seropositivity by the sVNT can be attributed to the decrease in levels of anti-nucleocapsid antibodies within 5–15 months of infection, whereas anti-spike antibody levels persist and gradually decline over time, indicating time as a strong influential factor [55]. So far, natural SARS-CoV-2 infection has not been reported in cheetahs. Seropositivity in cheetahs can be associated with their very low genetic diversity, which reduces the population’s resilience to environmental changes and increases susceptibility to diseases [56].

The high incidence of SARS-CoV-2 amongst white-tailed deer suggest that evaluating the susceptibility of other cervid species to SARS-CoV-2 could benefit disease management and intervention strategies. In a systematic surveillance study conducted in India, 33.33% (13/39) of buffaloes tested positive for SARS-CoV-2 RNA (delta B.1.1.617.2 variant) in nasal and/or rectal swab samples using RT-qPCR, suggesting that buffaloes are susceptible to SARS-CoV-2 [32]. Two experimental infection studies have evaluated the susceptibility of elk to the ancestral Wuhan-like and delta variants of SARS-CoV-2. A study conducted by Boggiatto et al. [30] infected seven North American elk calves and seven adults intranasally with the ancestral Wuhan-like isolate of SARS-CoV-2 (USAWA1/2020). Serum was evaluated using the RBD Inhibition ELISA assay (sVNT, GenScript Biotech, Piscataway NJ, USA) and showed that both calves and adult elk developed neutralizing antibodies as early as 7 days post-infection (dpi), which persisted for at least 21 days. Results were confirmed when serum was tested using a traditional VNT. Notably, the highest titers were detected in elk calves (1:256) at 14 dpi, whereas lower titers were observed in adult elk (1:16) at 7 dpi, indicating that the elk calves had a stronger neutralization response. The authors suggest that the lower neutralizing antibody responses in adult elk (older animals) might relate to pre-existing immunity from infection with related coronaviruses, which may explain a more limited response and a reduction in the amount of antigen available for de novo antibody responses. Additionally, viral RNA was detectable throughout the medial retropharyngeal lymph node in both calves and adult elk via RT-qPCR [30]. In a separate study by Porter et al. [29], inoculated weanling elk and mule deer were infected with the delta variant of SARS-CoV-2, B.1.617.2 via intranasal routes. This study demonstrated that inoculated mule deer shed infectious virus both orally and nasally. However, inoculated elk did not exhibit clinical signs, shed the virus, or transmit the virus to an in-contact elk. Neutralizing antibody responses were higher in mule deer, with peak neutralizing titers exceeding 1:1280 at 21 dpi compared to elk, which had peak neutralizing titers of 1:20 at 21 dpi. This experimental infection study demonstrated that elk have low susceptibility and mule deer have high susceptibility to the delta variant of SARS-CoV-2 [29].

Consistent with previous studies, our results indicated that 1.2% (7/575) of bison and 2.2% (4/180) of elk were seropositive in the sVNT, whereas 4.2% (24/575) of bison and 3.3% (6/180) of elk serum samples tested positive by the N-ELISA. The N protein is relatively conserved among coronaviruses, therefore higher seropositivity in N-ELISA compared to sVNT may result from potential cross-reactivity between SARS-CoV-2 and other betacoronaviruses found in cervid species. In this study, two bison samples and one elk sample contained virus-neutralizing antibodies via VNT, suggesting that bison and elk are susceptible to SARS-CoV-2. The seropositivity of BCoV in the bison and elk serum samples in our study were 7.7% (44/575) and 2.2% (4/180), respectively. When comparing results obtained from each assay, serum from one elk and one bison exhibited neutralizing antibodies in sVNT, VNT, and BCoV tests (Table 2), although three elk and five bison had tested positive for both the sVNT and BCoV tests. This was likely due to antigenic cross-reactions that might occur between BCoV and SARS-CoV-2. The high similarity between the spike protein epitopes of SARS-CoV-2 and bovine BCoV suggests that BCoV is one of the viruses most similar to SARS-CoV-2 [57]. 

## 5. Conclusions

Taken together, this study was one of the first to provide evidence of natural SARS-CoV-2 infection in bison, elk, and cheetahs in the USA. The presence of neutralizing antibodies in cheetahs, gorillas, lions, hippopotamuses, elk, and bison indicates their susceptibility to SARS-CoV-2 infection. It is crucial to monitor the impact of SARS-CoV-2 in bison and elk, both in the wild and in zoos, as well as other captive wildlife species. This will help better assess the reservoir potential of various animal populations for SARS-CoV-2, the risk of transmission in different settings, and the implications for both animal and human health.

## Figures and Tables

**Table 1:** Results of serological testing (sVNT, N-ELISAs, and VNT) of captive wild species.

Species	Taxonomy	Total Sample Tested	Geographical Location	Number of Positive Samples			
				sVNT	N-ELISA	VNT	VNT Titer
Elephant	*Loxodonta africana*	24	TN, CA, TX, OK	1			
Gray fox	*Urocyon cinereoargenteus*	29	IN, IA				
Llama	*Lama glama*	16	KS, MO				
Camel	*Camelus bactrianus*	13	KS				
Koala	*Phascolarctos cinereus*	7	CA, OH				
Alpaca	*Vicugna pacos*	6	KS, MA				
Rhinoceros	*Rhinoceros*	5	FL, MA, KS				
Lion	*Panthera leo*	4	WI, KS	2		2	1:2048
Addax	*Addax*	3	KS, MA				
Giraffe	*Giraffa camelopardalis*	3	KS, MA				
Panda	*Panda oleosa*	3	GA				
Tiger	*Panthera tigris*	3	FL				
Goral	*Naemorhedus goral bedfordi*	2	KS		1		
Porcupine	*Coendou roosmalenorum*	2	MO, KS				
Oryx	*Oryx beisa*	2	MA, KS				
Cheetah	*Acinonyx jubatus*	2	MA, KS	1		1	1:512
Zebra	*Pylopaguropsis zebra*	2	MA				
Hippopotamus	*Hippopotamus amphibius*	1	MA	1		1	1:8
Wallaby	*Petrogale wilkinsi*	1	KS				
Bearcat	*Arctictis binturong*	1	KS				
Lynx	*Lynx*	1	KS				
Chevrotain	*Tragulidae*	1	TX				
Mink	*Neogale vison*	1	KS				
Sea lion	*Otariidae*	1	PA				
Hylobates lar	*Hylobates lar*	1	KS				
Antelope	*Hippotragus equinus*	1	FL				
Caribou	*Rangifer tarandus*	1	MO				
Bobcat	*Lynx rufus*	1	KS				
Hyena	*Crocuta crocuta spelaea*	1	MA				
Impala	*Aepyceros melampus*	1	MA				
Primate	*Haplorrhini*	1	CA				
Ferret	*Mustela putorius furo*	1	WA				
Bear	*Ursus* sp.	1	GA				
Buck	*Hippotragus leucophaeus*	1	FA				
Gorilla	*Gorilla gorilla*	1	MA	1	1	1	1:128

**Table 2:** Comparison and concordance of elk and bison serums evaluated by sVNT, VNT, and BCoV tests.

Species	Taxonomy	Testing Results			
		sVNT	VNT	BCoV	Total (Numbers)
**Elk**	*Cervus canadensis nelsoni*	Pos *	Pos	Pos	1
		Pos	Neg **	Pos	3
		**sVNT**	**VNT**	**BCoV**	**Total (Numbers)**
**Bison**	*Bison bison*	Pos	Pos	Pos	1
		Neg	Neg	Neg	19
		Neg	Neg	Pos	38
		Pos	Pos	Neg	1
		Pos	Neg	Pos	5

* Pos: Positive; ** Neg: Negative.

## Data Availability

The original contributions presented in the study are included in the article, further inquiries can be directed to the corresponding author.
